# Transforming Dental Care in Saudi Arabia: Challenges and Opportunities

**DOI:** 10.7759/cureus.54282

**Published:** 2024-02-16

**Authors:** Omar S Almajed, Alhareth Aljouie, Rahaf Alghamdi, Faris N Alabdulwahab, Malak T Laheq

**Affiliations:** 1 Pediatric Dentistry, Imam Abdulrahman Bin Faisal University, Dammam, SAU; 2 Dental Public Health, King’s College London, London, GBR

**Keywords:** socio-cultural factors, special healthcare needs, dental care barriers, technology integration, accessibility, challenges, evolution, oral health, saudi arabia, dental healthcare

## Abstract

This comprehensive review examines the dental healthcare system in Saudi Arabia, tracing its evolution, current status, and persisting challenges. The system has evolved significantly due to government initiatives and technological integration, yet it grapples with issues like professional shortages, financial constraints, and disparities in access. Despite improvements in accessibility, geographic and socio-economic disparities persist, and oral health promotion remains limited. The integration of technology faces resistance, and specialized care for people with disabilities requires targeted strategies. Cultural and social factors influence oral health, and the system's response to the COVID-19 pandemic showcases adaptability and patient-centered approaches. Addressing these challenges is crucial for ensuring equitable and effective oral health service delivery in Saudi Arabia.

## Introduction and background

This review aims to comprehensively examine the dental healthcare system in Saudi Arabia, highlighting its evolution, current state, and the multifaceted challenges it faces. In recent decades, Saudi Arabia has seen significant transformations in its dental healthcare landscape, driven by government prioritization and strategic health service development. However, this progress has not been without its challenges.

The Saudi dental healthcare system, characterized by a blend of government and private sector involvement, has improved health outcomes but continues to grapple with issues such as professional shortages, financial constraints, and high demand due to free services [1]. Accessibility to dental services remains a critical concern, with many citizens preferring private dental services over government-provided care, often perceived as superior [2].

Primary healthcare centers, pivotal in providing oral healthcare, encounter challenges like high costs in private clinics and dentist unavailability, impacting service delivery [3]. Furthermore, oral hygiene practices among the Saudi population are not widespread, and the utilization of healthcare for oral disease prevention is limited, underscoring the need for enhanced oral health promotion [4].

The integration of technology, such as electronic dental record (EDR) systems, has faced resistance due to factors like staff reluctance, a lack of technical skills, and usability issues [5]. Specialized care groups, including individuals with special healthcare needs and autistic children, face unique challenges in accessing dental care, highlighting the need for targeted strategies to overcome barriers like physical accessibility, affordability, and provider knowledge gaps [6,7]

This review will delve into these aspects, offering a detailed analysis of the current state of the dental healthcare system in Saudi Arabia, its developmental trajectory, and the challenges that need addressing to ensure effective and equitable oral health service delivery.

## Review

Materials and methods

This narrative review of the dental healthcare system in Saudi Arabia involved a comprehensive literature search across multiple databases, including PubMed, Google Scholar, and Chat.Consensus.App plugin. The search strategy was designed to identify peer-reviewed articles, clinical trials, and retrospective studies published within the last decade. Key search terms included "dental healthcare system in Saudi Arabia," "oral health services in Saudi Arabia," "challenges in dental care in Saudi Arabia," and related terms.

The inclusion criteria were meticulously defined to ensure the relevance and specificity of the review. Selected articles that need to be published in English focus on the dental healthcare system in Saudi Arabia and cover aspects such as policy development, service accessibility, technological integration, and challenges faced by special patient groups. Essential metrics for inclusion included data on system effectiveness, patient outcomes, and healthcare delivery models. We excluded studies that did not specifically address the dental healthcare system in Saudi Arabia, were published more than ten years ago (except for historical context), or were non-peer-reviewed literature.

The analysis of the dental healthcare system in Saudi Arabia involved a methodical process of data extraction and analysis, focusing on the system's development, challenges, policy implications, service accessibility, and patient outcomes. In selecting studies for review, priority was given to their relevance and the depth of insight they provided into the complexities of the dental healthcare landscape, including its evolution, present challenges, and potential future directions. The research did not follow a strict quality assessment protocol; instead, it emphasized the significance and reliability of each study's findings. This approach, combined with a qualitative synthesis to compare and contrast various studies, was designed to offer a comprehensive and insightful overview of the dental healthcare system in Saudi Arabia, ensuring the review included the most impactful and credible evidence available.

Review

Evolution of the Dental Healthcare System in Saudi Arabia

The dental healthcare system in Saudi Arabia has experienced a significant transformation over recent decades, evolving from a focus on basic dental needs to providing comprehensive and specialized services. This evolution has been driven by various factors, including government initiatives, economic growth, and an increased emphasis on healthcare education and promotion [8].

A key development in this transformation has been the integration of technology, particularly the adoption of EDR. This move towards digitalization aims to enhance the efficiency and quality of dental services, although it has faced challenges such as staff resistance, a lack of technical skills, and usability issues [5].

Another important aspect of the system's evolution is the increased focus on providing dental care to individuals with special healthcare needs. Efforts have been made to understand and address barriers faced by these groups, including issues related to physical accessibility, affordability, and provider knowledge gaps [6,7].

Amid these advancements, Vision 2030 emerges as a pivotal government initiative aiming to further revolutionize the healthcare sector, including dental care. Launched by the Saudi government, Vision 2030 seeks to diversify the nation's economy and improve the quality of life for its citizens by setting ambitious healthcare goals. These include expanding access to care, enhancing the quality of healthcare services, and promoting preventive health measures. Vision 2030's emphasis on healthcare innovation and technology integration aligns with the ongoing efforts to modernize the dental healthcare system, promising a future where dental care is more accessible, efficient, and patient-centered. A study by Althumairy highlights the role of Vision 2030 in aiming to improve the quality of healthcare while maintaining the efficacy of spending, underscoring its potential impact on the dental healthcare sector [9,10].

Despite these advancements, the Saudi dental healthcare system continues to grapple with challenges like professional shortages, financial constraints, and high demand due to free services [1]. Additionally, there is a pressing need for enhanced oral health promotion, as practices like regular brushing and flossing are not widespread among the population [4].

In summary, the evolution of the dental healthcare system in Saudi Arabia reflects a journey toward modernization and inclusivity, significantly influenced by Vision 2030. Significant progress has been made, but ongoing efforts are essential to overcome existing challenges and ensure effective and equitable oral health service delivery for the entire population.

Accessibility to Dental Services

While the expansion of dental facilities has undoubtedly improved access to dental services in Saudi Arabia, challenges related to accessibility persist. Many citizens still prefer private dental services over government-provided care, viewing them as superior [2]. This preference for private care can create disparities in access to dental services, particularly for those who cannot afford private healthcare.

Additionally, the distribution of dental clinics and professionals across the country is not uniform. Urban areas tend to have better access to dental services, while rural and remote regions may still face challenges in accessing quality oral healthcare [3]. This geographic disparity in access needs to be addressed through strategic planning and the establishment of dental clinics in underserved areas.

To improve accessibility, the Saudi government could consider initiatives such as mobile dental clinics to reach remote communities. Telehealth and tele-dentistry solutions can also be explored to provide consultations and follow-up care for patients in areas with limited access to dental facilities [5]. These technological advancements can bridge the gap in dental service availability.

Furthermore, specialized care groups, including individuals with special healthcare needs and autistic children, face unique challenges in accessing dental care. These challenges highlight the need for targeted strategies to overcome barriers like physical accessibility, affordability, and provider knowledge gaps [6,7].

In conclusion, while there have been improvements in the dental healthcare system in Saudi Arabia, significant efforts are still needed to ensure equitable and effective access to dental services for all citizens, particularly in rural areas and for special needs populations.

Oral Health Promotion and Prevention

One of the critical aspects of a robust dental healthcare system is the promotion of oral health and the prevention of dental diseases. However, in Saudi Arabia, oral hygiene practices among the population are not widespread, and the utilization of healthcare for oral disease prevention is limited [4]. This highlights the need for comprehensive oral health promotion strategies.

Public education campaigns can play a pivotal role in raising awareness about the importance of oral hygiene and regular dental check-ups. Schools, community centers, and healthcare facilities can serve as platforms for disseminating information about proper oral care practices. Collaborative efforts between healthcare authorities and educational institutions can facilitate the integration of oral health education into the curriculum.

Furthermore, preventive programs, such as community water fluoridation and dental sealant programs, can be implemented to reduce the incidence of dental caries. These initiatives have proven effective in preventing dental diseases, especially among children. Studies have shown that the prevalence and severity of dental caries among schoolchildren in the Gulf Cooperation Council area, including Saudi Arabia, are significant, indicating the need for systematic approaches to preventive oral care programs [11].

Additionally, the effectiveness of different modes of school dental health education, such as drama, games, and flashcards, has been explored, with findings suggesting that child-friendly modes of delivery are more impactful in improving oral health status [12]. Moreover, there is evidence that school-based oral health promotion programs involving teachers and parents can lead to improved oral health knowledge and behavior [13].

In conclusion, to address the limited utilization of healthcare for oral disease prevention in Saudi Arabia, a multi-faceted approach involving public education campaigns, school-based programs, and community preventive initiatives is essential. These strategies should aim to improve oral hygiene practices and increase awareness of the importance of regular dental care.

Integration of Technology in Dental Care

The integration of technology, particularly electronic dental record systems, has the potential to enhance the efficiency and quality of dental care in Saudi Arabia; however, as evidenced by the findings in Table 1, this adoption has encountered notable resistance stemming from factors such as staff reluctance, insufficient technical skills, and usability challenges [5].

**Table 1:** Challenges and solutions for technology integration in dental care

Challenge	Potential Solutions
Staff resistance	Training and education programs for staff
Lack of technical skills	Skill development workshops and courses
Usability issues	User-friendly software and interface design
Cost barriers	Government subsidies or incentives for technology

A national cross-sectional survey investigating the status of digital dental technology (DDT) adoption in Saudi Arabian undergraduate dental education revealed that while 64.4% of dental schools implemented DDT in their curricula, challenges such as lack of expertise, untrained faculty and staff, and cost were significant barriers. This indicates a need for a more comprehensive integration of digital components in dental education and patient care treatment to align with Saudi Vision 2030 for healthcare digitization [14].

Innovations like flexible and biocompatible high-performance solid-state micro-batteries for implantable orthodontic systems have also been developed. These technologies, integrated into smart dental braces, could serve in faster bone regeneration and enhanced enamel healthcare protection, reducing overall healthcare costs [15].

Furthermore, the development of a dental clinic database system for a general tertiary hospital in Riyadh highlights the importance of integrating information technology into dental care. This project, based on the experience and knowledge of a practicing dentist, aimed to build a database system for the storage, organization, and recovery of data, demonstrating the potential benefits of technology in improving dental care delivery [16].

In conclusion, while there is a clear trend towards integrating technology in dental care in Saudi Arabia, challenges such as resistance from staff, a lack of technical skills, and cost barriers need to be addressed. Training programs and support for dental staff, along with user-friendly and intuitive software, are essential to promoting the successful integration of technology in dental care.

Specialized Dental Care for Persons With Disabilities

Specialized dental care for persons with disabilities in Saudi Arabia is a critical area that necessitates focused attention, as underscored in Table 2, which delineates the access barriers encountered by persons with disabilities and children with special healthcare needs (CSHCN). Research has pinpointed various obstacles and challenges these groups face in accessing dental care.

**Table 2:** Access barriers for persons with disabilities

Vulnerable population	Access barriers	Potential solutions
Individuals with special healthcare needs	Lack of specialized care, transportation issues, clinic environment	Specialized dental clinics, mobile clinics, caregiver support, continuing education for general practitioners in special care dentistry
Children with special healthcare needs	Fear of the dentist, child uncooperativeness, treatment costs	Dentist training and education, financial assistance programs, specialist programs to train dentists in special care dentistry

A study conducted in Qatif, Saudi Arabia, concentrated on persons with disabilities and reported significant difficulties in accessing dental care as perceived by caregivers. The primary barriers included limited availability of caregivers' time, clinic environments that are not accommodating, transportation challenges, the medical or health status of the individual, and geographically remote dental clinics. This study emphasizes the urgent need for more accessible and accommodating dental services for people with disabilities [6].

Another investigation in Jeddah, Saudi Arabia, explored dental care access among CSHCN. It was discovered that only a minor fraction of caregivers routinely visited the dentist for their CSHCN. The most frequently cited barriers were fear of the dentist, child uncooperativeness, and the costs associated with treatment. This study highlights the critical need for dentists to undergo additional training and education to enhance access to dental care for CSHCN [17].

Moreover, a comprehensive review by Asiri et al. synthesized evidence regarding the current status, trends in oral health behaviors, and oral healthcare utilization among persons with disabilities in Saudi Arabia. It concluded that individuals with disabilities have limited access to oral health care, poor oral health status, and a general lack of awareness regarding oral health. The review also identified several barriers to dental treatment, including distance, lack of trained specialists, and costs of treatment, underscoring the need for nationwide surveys to fully understand the extent of oral health inequities among individuals with disabilities [18].

In summary, the research indicates that people with disabilities in Saudi Arabia, including children with special healthcare needs, encounter significant barriers to accessing dental care. Overcoming these challenges necessitates concerted strategies aimed at improving physical accessibility and affordability and enhancing the knowledge and skills of dental care providers for these groups.

Public education campaigns have been shown to significantly impact oral health awareness and practices among schoolchildren. For instance, a study conducted by Halawany et al. demonstrated the effectiveness of oral health education interventions in improving oral hygiene knowledge and practices among female primary school children in Riyadh, Saudi Arabia. This underscores the importance of integrating oral health education into school curricula to foster better oral hygiene practices from a young age [19].

Cultural and Social Factors Influencing Oral Health

The influence of cultural and social factors on oral health in Saudi Arabia, as elucidated in Table 3, is a multifaceted phenomenon supported by extensive research. For instance, a study conducted on Saudi Arabian children with sensory impairments attending special schools in Riyadh revealed that gender and social class background significantly influenced the rating and self-perception of malocclusion among the children. This finding suggests that cultural perceptions of dental aesthetics and the need for orthodontic treatment vary across different social strata, highlighting the role of socio-cultural factors in shaping attitudes toward dental care [20].

**Table 3:** Cultural and social factors influencing oral health

Factor	Influence on oral health
Gender	Different perceptions of dental aesthetics
Social class background	Varied self-perception of malocclusion across social strata
Socioeconomic status	Influence on oral health practices and outcomes
COVID-19 impact	Changes in health-seeking behavior during the pandemic

In addition to perceptions and attitudes, the Oral Health Impact Profile (OHIP) has been utilized to assess oral health-related quality of life among adults in Saudi Arabia. The adaptation and validation of the Arabic version of OHIP for the Saudi context indicate its suitability for assessing oral health-related quality of life, reflecting the cultural relevance of oral health outcomes [21].

Parental education and socioeconomic status also play a crucial role in influencing oral health. A study in the eastern province of Saudi Arabia found that higher education levels and higher monthly incomes among parents were significantly associated with a lower prevalence of decayed teeth in their children. This association underscores the impact of socio-economic factors on oral health practices and outcomes, emphasizing the need for targeted oral health education and intervention programs that consider these socio-economic disparities [22].

Furthermore, the COVID-19 pandemic has had a significant impact on oral health behaviors and practices. A study assessing the impact of the pandemic lockdown on oral health and behavior change among children in the eastern province of Saudi Arabia demonstrated considerable alterations in children's dental health. Changes in daily routines and access to dental care during the lockdown period led to significant behavioral changes, reflecting the influence of external environmental factors on oral health [23].

These studies collectively highlight the complex interplay of cultural, social, and economic factors in shaping oral health behaviors and outcomes in Saudi Arabia. They underscore the importance of considering these factors in designing and implementing oral health promotion and intervention programs to effectively address the unique needs of the Saudi population.

Healthcare System Response to Pandemics and Emergencies

The healthcare system in Saudi Arabia, particularly in the field of dentistry, has demonstrated commendable adaptability and innovation in response to pandemics and emergencies, such as the COVID-19 crisis. The implementation of teledentistry services during the pandemic is a prime example of this adaptability. A study conducted in 2022 highlighted that while the utilization of teledentistry services among dental care seekers during COVID-19 was relatively low, the existing technological infrastructure in Saudi Arabia provided a solid foundation for these services. The study emphasized the potential of teledentistry in offering dental diagnosis, consultations, monitoring, and triaging of dental cases, both emergency and non-emergency, during the pandemic [24].

Moreover, the response to the pandemic in the dental sector also involved a significant shift in health-seeking behaviors. During the initial phase of the COVID-19 pandemic, dental clinics in Saudi Arabia were recommended to close except for emergency dental care. This led to changes in the public's decisions to seek dental care, with a study showing that social determinants played a crucial role in these decisions. The study found that factors such as gender, previous dental visits, and living in metropolitan regions influenced the likelihood of seeking emergency dental care during the pandemic. This indicates a nuanced understanding of and response to the pandemic within the dental healthcare community [25].

The Saudi dental healthcare system's response to the COVID-19 pandemic reflects its strength and resilience. The swift adaptation to new modes of service delivery, like teledentistry, and the careful consideration of social determinants in healthcare-seeking behavior demonstrate a proactive and patient-centered approach. These efforts not only ensured continued access to essential dental services during a critical time but also laid the groundwork for future advancements in dental healthcare delivery in the face of emergencies.

## Conclusions

The dental healthcare system in Saudi Arabia, while evolving with technological advancements and specialized care, still faces challenges such as professional shortages, financial constraints, and access disparities, especially in rural areas and among persons with disabilities. Despite improvements, issues like limited oral health promotion and the impact of cultural and social factors on oral health persist. The COVID-19 pandemic has underscored the system's adaptability through teledentistry and altered health-seeking behaviors. To enhance oral health service delivery, there is a pressing need for strategic planning and policy reforms focusing on equitable access, addressing professional shortages, and expanding oral health promotion. In essence, while progress has been made, significant efforts are required to ensure all individuals receive equitable and effective dental care in Saudi Arabia.
